# Brachial Artery Entrapment Due to Supracondylar Humerus Fracture—Two Case Reports

**DOI:** 10.3390/children13070938

**Published:** 2026-07-17

**Authors:** Marko Radman, Ana Bosak Versic

**Affiliations:** 1 Department of Pediatric Surgery, Clinical Hospital Center Rijeka, Vjekoslava Dukića 7, 51000 Rijeka, Croatia; marko.radman@medri.uniri.hr; 2 Department of Pediatric Surgery, Faculty of Medicine, University of Rijeka, Istarska 43, 51000 Rijeka, Croatia

**Keywords:** brachial artery, entrapment, pediatric trauma, supracondylar fracture, vascular injury

## Abstract

**Highlights:**

**What are the main findings?**

- Brachial artery entrapment is a rare but serious complication of pediatric supracondylar humerus fracture.
- Early surgical exploration is crucial when vascular status does not improve after reduction.

**What are the implications of the main findings?**

- Careful neurovascular assessment is essential in all pediatric elbow fractures.
- Delayed intervention may result in permanent neurovascular impairment.

**Abstract:**

**Background/Objectives:** Supracondylar humerus fractures are among the most common pediatric fractures and may be associated with neurovascular complications. Brachial artery entrapment is a rare but limb-threatening condition requiring urgent recognition and management. **Methods:** We present two pediatric cases of brachial artery entrapment associated with displaced supracondylar humerus fractures. Clinical presentation, imaging findings, surgical management, and outcomes were analyzed. **Results:** Both patients presented with absent radial pulse following trauma. Initial fracture reduction and fixation did not restore vascular flow. CT angiography confirmed brachial artery compromise. Surgical exploration with arterial decompression resulted in restoration of perfusion. One patient achieved full recovery, while the other developed severe neuropathy with partial recovery after rehabilitation. **Conclusions:** Brachial artery entrapment should be suspected in cases of persistent vascular compromise after fracture reduction. Prompt surgical intervention and thorough perioperative neurovascular assessment are essential to prevent long-term morbidity.

## 1. Introduction

Supracondylar fractures of the humerus are common elbow fractures in children [1]. They account for the majority of pediatric elbow fractures, with a peak incidence between 5 and 8 years of age [2,3]. Boys are usually more affected than girls, but in some series a higher incidence in girls has been reported [4]. Regarding the mechanism of injury, there are two types of supracondylar fractures: the flexion type, which comprises less than 1 percent, and the extension type, which accounts for 99 percent of all cases [2]. Based on the degree of displacement, there are four Gartland types of fractures [1]. Type I fractures are nondisplaced. Type II fractures are displaced anteriorly, but the posterior cortex of the humerus remains intact. Type III fractures are completely displaced with no cortical contact. Type IV fractures have an incompetent periosteal hinge circumferentially and are unstable in both flexion and extension [5,6].

In the pediatric population, closed reduction and pinning with K-wires is the treatment of choice for Gartland type II, III, and IV fractures, while open fractures or neurovascular compromise may indicate the need for open reduction. Severe displacement is usually associated with complications, including neurovascular injuries, nonunion, or compartment syndrome. Neurovascular injury occurs in up to 49% of severely displaced fractures, with vascular compromise reported in 3–19% of cases [7]. Possible vascular injuries include complete transection, kinking of the brachial artery with reduced flow, thrombosis, intimal tear, arterial contusion or spasm, entrapment of the vessel within the fracture site, or traumatic aneurysm of the brachial artery with subsequent thrombus formation [8]. Although rare, arterial injury due to entrapment at the fracture site is a surgical emergency that requires prompt diagnosis and intervention [9].

There are controversies regarding the clinical presentation and treatment of supracondylar fractures with vascular compromise. “Pulseless pink hand,” in which some surgeons choose to wait and see rather than explore the neurovascular bundle, remains a clinical decision based on the surgeon’s preference and experience [10,11,12]. In contrast, “pulseless white hand” presents a clear clinical indication to explore the neurovascular bundle [7,10]. Here we present two patients with brachial artery entrapment due to supracondylar humerus fracture.

## 2. Materials and Methods

This study presents two case reports of pediatric patients treated for supracondylar humerus fractures complicated by brachial artery entrapment. Clinical data, imaging findings, surgical procedures, and postoperative outcomes were reviewed retrospectively. Ethical approval was obtained in accordance with institutional guidelines.

## 3. Results

### 3.1. Case 1

A 6-year-old boy presented to our emergency clinic after a direct fall onto his left elbow. The incident occurred 3 h before his arrival at our institution. Initial examination revealed a severely displaced elbow with no radial pulse but normal capillary reperfusion time (pulseless, pink hand). Radiographs in two planes showed a posteriorly displaced supracondylar fracture, Gartland type III. Immediate surgical intervention was indicated. Closed reduction of the fracture and percutaneous fixation with two K-wires were performed under general anesthesia (Figure 1).

Afterward, the radial pulse was still absent, but finger pulse oximetry showed 100% oxygen saturation. As advised by the vascular surgeon who was immediately consulted, emergency CT angiography (Figure 2) of the left arm was performed, which showed discontinuity of contrast in the terminal part of the brachial artery. Exploration of the neurovascular bundle via an anterior cubital approach was indicated and revealed entrapment of the brachial artery at the fracture site. Mobilization, decompression, and heparinization of the artery were carried out. After the procedure, a strong pulse was noted in the radial and ulnar arteries. Satisfactory intraoperative ultrasound Doppler flow was observed. The radial pulse was achieved 6 h after hospital admission, 9 h after the incident.

Postoperatively, the boy was admitted to the pediatric ICU for 24 h of observation. His left arm was placed in an elbow cast. Enoxaparin was administered during the period of immobilization, resulting in a hospitalization time of 10 days, during which follow-up Doppler ultrasound showed satisfactory blood flow in the left arm. Immobilization lasted 14 days, after which follow-up radiographs showed strong callus formation (Figure 3). The K-wires were removed, and physical therapy was started. Four months after the injury, complete function of the arm and hand had returned.

### 3.2. Case 2

A 7-year-old girl was admitted to our emergency department 6 h after falling from a trampoline onto her left hand. The affected upper extremity was pale, with an absent radial pulse and prolonged capillary refill time (pulseless white hand). No clinical signs of compartment syndrome were present at initial examination. Palpation demonstrated soft, compressible compartments without evidence of elevated compartment pressure. Two-plane radiographs showed a posterolaterally displaced supracondylar fracture of the humerus, Gartland type III (Figure 4). She was taken urgently to the operating room, where closed reduction and fixation with two K-wires were performed under general anesthesia. As there was no improvement in skin color or capillary refill time, and the radial pulse remained absent, emergency CT angiography of the left upper limb was performed. The CT showed discontinuity of contrast in the terminal part of the brachial artery, and open exploration of the neurovascular bundle via an anterior cubital approach was performed. The brachial artery was entrapped at the fracture site. Mobilization and decompression of the brachial artery produced a satisfactory vascular response, with immediate return of limb color, a palpable radial pulse, and normal capillary reperfusion time (Figure 5). The radial pulse was restored 4 h after hospital admission, 10 h after the incident. Because of swelling and to prevent compartment syndrome, the wound was left open. On the second postoperative day, total loss of sensation and motor function in the left forearm was noted, indicating injury to the median, ulnar, and radial nerves. On the fourth postoperative day, when the swelling had subsided, the wound was treated with negative pressure therapy. She was discharged from the hospital on the 18th day, after removal of the K-wires and splint. EMNG showed severe post-traumatic neuropathy of the left forearm. Follow-up ultrasound Doppler showed normal blood flow. After 6 months of intensive physical therapy, she regained only partial function of the hand, and follow-up EMNG showed partial neural recovery.

## 4. Discussion

Gartland type III supracondylar fractures are commonly associated with primary neurovascular lesions [13,14,15,16]. Posteromedial displacement (Gartland type III A) is associated with radial nerve injury, and posterolateral displacement (Gartland type III B) is associated with median nerve injury. In contrast, brachial artery injury can occur with both types of displacement without clear predominance [17,18]. The initial treatment of pediatric supracondylar fractures with absent radial pulse and a cold, pale hand is closed reduction and fixation with K-wires. If reduction fails to restore vascular stability of the hand, open reduction and vascular exploration are recommended [19]. In contrast, the management of children with absent radial pulse but a well-perfused hand remains controversial [19,20,21], although most authors suggest immediate open surgery and arterial exploration [22].

Our two cases illustrate both clinical scenarios. In our first patient, the hand remained pulseless but pink after primary reduction and fixation. Because vascular status failed to return to stability, we proceeded with open vascular exploration. Intraoperative findings confirmed brachial artery entrapment, and surgical release restored vascular flow and resulted in excellent functional recovery. This case supports the view that persistent absence of the radial pulse following reduction should raise suspicion of an underlying mechanical arterial lesion requiring surgical exploration rather than a “watchful waiting” approach.

The second patient presented with a pulseless, poorly perfused hand, necessitating urgent vascular exploration after unsuccessful closed reduction. In contrast to the first patient, the clinical outcome was less favorable. Although a direct causal relationship cannot be definitively established, the prolonged interval between injury and definitive surgical treatment, exceeding 8 h, together with progressive soft-tissue swelling, likely contributed to the severity of neurovascular injury. Part of this delay resulted from extended evaluation and transfer from the referring hospital. Despite successful restoration of arterial flow, the patient developed severe radial, median, and ulnar neuropathies with marked impairment of upper-limb function, achieving only partial neurological recovery after intensive rehabilitation. Interestingly, several studies have reported no significant difference in perioperative complication rates between early (≤8 h) and delayed (>8 h) operative treatment [23]. Nevertheless, our experience suggests that prolonged ischemia and increasing soft-tissue edema may worsen neurological injury in individual patients, even when vascular reconstruction is ultimately successful.

Current evidence does not support routine CT angiography in the management of these injuries. In isolated supracondylar fractures, vascular injury is almost invariably located at the fracture site, and additional imaging rarely alters surgical management. Instead, angiography or other vascular imaging modalities may unnecessarily delay fracture reduction and vascular decompression, thereby increasing the risk of ischemic complications [24,25]. In both of our patients, CT angiography was performed at the request of the on-call vascular surgeon. Although these studies confirmed vascular compromise, they did not change the subsequent surgical strategy and likely prolonged the time to definitive treatment.

Mechanical entrapment of the brachial artery may result from interposition of perivascular soft tissues, tethering within the fracture site, or compression by a sharp bony fragment. Importantly, these lesions may remain clinically occult because vascular insufficiency may become apparent only in specific elbow positions, particularly full flexion or extension [9,26,27]. Consequently, reliance on pulse palpation alone may fail to identify dynamic arterial obstruction.

Given the variability of clinical presentation, the potential severity of neurovascular injury, and the inherent challenges of neurovascular examination in young children, a standardized neurovascular assessment is essential [9]. Careful documentation of vascular perfusion, along with motor and sensory function of the median, radial, and ulnar nerves, should be performed before reduction, immediately after fixation, and repeatedly during the postoperative period. Postoperative assessment should include evaluation of distal perfusion throughout the full range of elbow motion, as dynamic brachial artery entrapment may become evident only during flexion or extension [9]. In addition, associated ipsilateral upper-limb injuries should be actively excluded, and patients should be closely monitored for the development of compartment syndrome, particularly in the presence of marked swelling, extensive ecchymosis, or persistent vascular compromise [2]. In our second patient, the severity of soft-tissue swelling and concern for impending compartment syndrome prompted us to leave the wound open. Although compartment syndrome did not subsequently develop, this approach allowed safe postoperative monitoring while minimizing the risk of elevated compartment pressure.

## 5. Conclusions

Our two cases demonstrate a rare but clinically important neurovascular complication—segmental entrapment of the brachial artery—associated with severely displaced pediatric supracondylar humeral fractures. Although vascular compromise is a well-recognized complication of these injuries, true segmental entrapment of the brachial artery within the fracture site remains uncommon and may be easily overlooked.

The second case, involving a 7-year-old girl, further illustrates the potential consequences of delayed surgical intervention. A delay of more than 8 h from injury to definitive treatment, combined with progressive soft-tissue swelling and an increasing risk of compartment syndrome, may have contributed to secondary injury of the adjacent radial, ulnar, and median nerves. Although a direct causal relationship cannot be definitively established, this case highlights how prolonged vascular compromise and increasing tissue edema may exacerbate neurological injury and negatively affect functional recovery. In contrast, the first patient, who underwent earlier surgical intervention, achieved complete neurological recovery, emphasizing the potential importance of timely recognition and management of neurovascular compromise.

These cases underscore the necessity of meticulous, standardized neurovascular assessment throughout the entire course of treatment. Preoperatively, a systematic evaluation of peripheral perfusion, vascular status, and motor and sensory function of the median, radial, and ulnar nerves should be performed and carefully documented to facilitate appropriate surgical planning and timely intervention. Intraoperatively, thorough exploration of the neurovascular bundle should be considered whenever vascular injury or arterial entrapment is suspected, particularly in patients presenting with a pulseless extremity or persistent vascular insufficiency following fracture reduction. Postoperatively, serial neurovascular examinations should be routinely performed, including repeated assessment of distal perfusion, nerve function, pain, compartment syndrome, and the full range of elbow motion, allowing early recognition of evolving complications and prompt intervention when necessary.

Given the rarity of this injury pattern, reporting additional cases and accumulating clinical experience will be essential to improve understanding of its pathophysiology, optimize diagnostic and treatment algorithms, and ultimately reduce the risk of long-term neurovascular deficits and functional impairment in children with supracondylar humeral fractures.

## Figures and Tables

**Figure 1:**
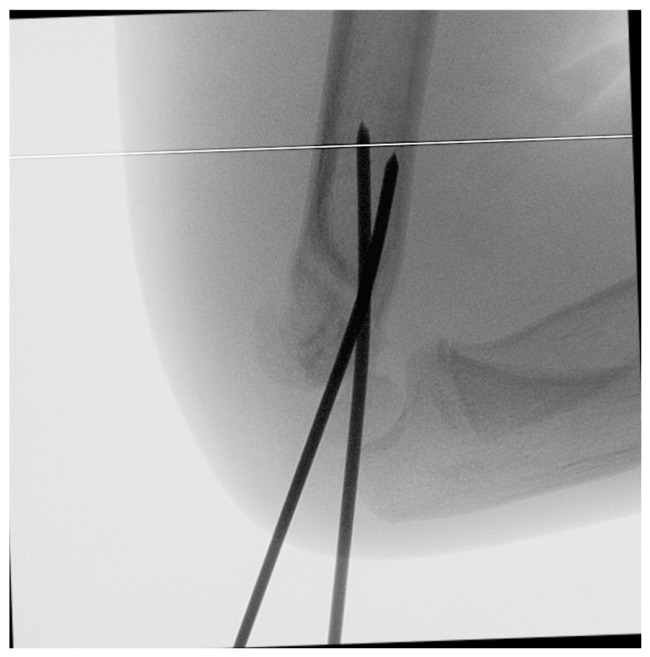
Lateral projection, intraoperative, showing reduced fracture and K-wires placed.

**Figure 2:**
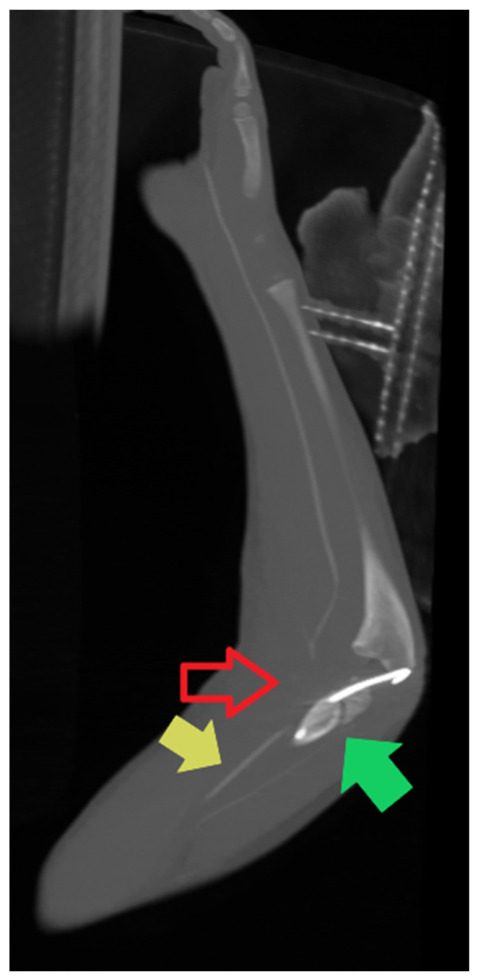
CT angiography showing a contrast defect in the brachial artery at the level of the fracture site (red arrow). The yellow arrow indicates the brachial artery, and the green arrow indicates the fracture with K-wire placement.

**Figure 3:**
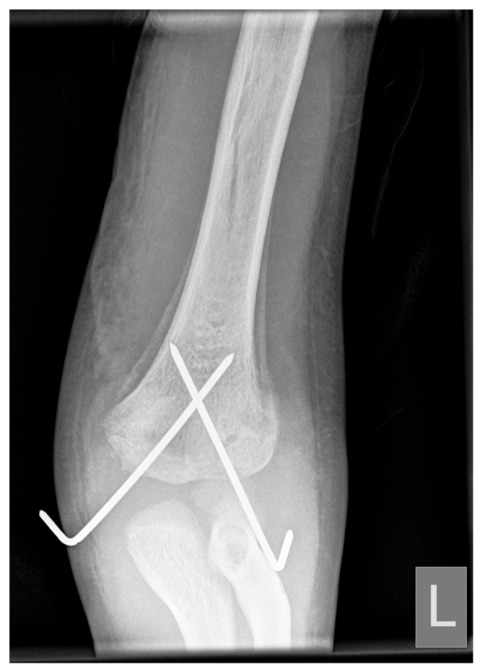
Anteroposterior projection after 2 weeks showing strong callus formation.

**Figure 4:**
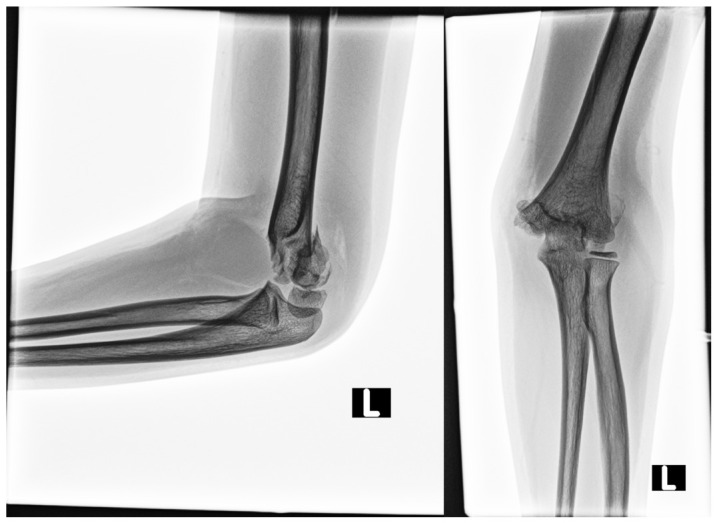
X-ray images of left elbow, showing supracondylar fracture type Gartland III.

**Figure 5:**
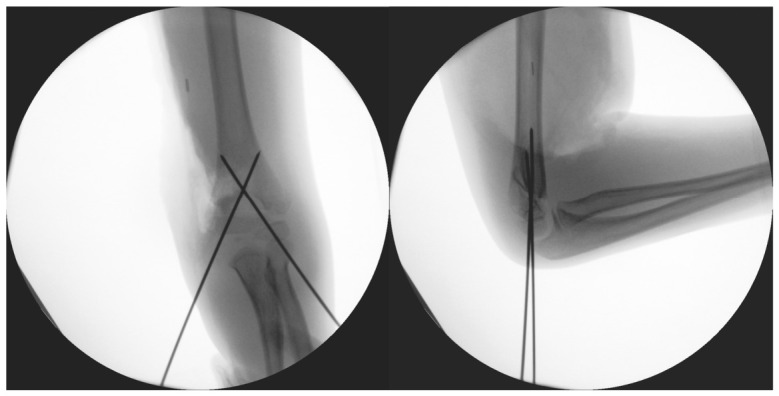
Intraoperative X-ray images showing fixation with K-Wires.

## Data Availability

Data are available upon reasonable request.

## Abbreviations

The following abbreviations are used in this manuscript:

CT	Computed tomography
ICU	Intensive care unit
EMNG	Electromyoneurography
